# “Evaluation of Silver Diamine Fluoride Modified Atraumatic Restorative Treatment (SMART) on hypomineralized first permanent molar”- a randomized controlled clinical study

**DOI:** 10.1186/s12903-024-04860-z

**Published:** 2024-10-04

**Authors:** Aya Ehab Saad, Ashraf Yassin Alhosainy, Abeer M. Abdellatif

**Affiliations:** 1 Pediatric Dentistry, Department of Pediatric Dentistry and Public Health, Faculty of Dentistry, Delta University, International Coastal Rd, Al Hafir WA Al Amal, Al Satamoni, Dakahlia Governorate, 7730103 Egypt; 2 Pediatric Dentistry, Department of Pediatric Dentistry and Dental Public Health, Faculty of Dentistry, Mansoura University, Mansoura, Egypt

**Keywords:** Silver diamine fluoride, Hypomineralized first permanent molars, Glass ionomer restorations

## Abstract

**Background:**

Restoring first permanent molars affected with molar incisor hypomineralization (MIH) is challenging. Focusing on improving the quality of life for children affected by MIH, at least until the complete eruption of first permanent molars to receive full coverage, to decrease the hypersensitivity and to be able to perform proper oral hygiene measures, the purpose of this study was to compare silver modified atraumatic restorative technique (SMART) versus the conventional restoration and fluoride varnish application on moderate to severe hypomineralized molars. The comparison considered the restoration survival, hypersensitivity, and digital surface area changes after one year follow up.

**Methods:**

Twenty-eight children were selected (20 girls and 8 boys) with at least 2 MIH molars with the same defect severity. The study comprised 2 groups; MOD group (moderate hypomineralized molar severity) and SEV group (Severe hypomineralized molar severity) (*n* = 28 tooth). Each group was further subdivided into 2 subgroups according to the technique of restoration: SMART subgroup and CONV subgroup (high viscosity glass ionomer restoration and fluoride varnish application) (each = 14 tooth). Evaluation was done in terms of the restoration survival (6 months and 12 months), hypersensitivity at 1 weak, 6 months and 12 months and occlusal surface area changes at 12 months). Professional Fluoride varnish application and home prophylaxis using MI paste were the protocol for each child patient.

**Results:**

There was no significant difference between the 4 subgroups, regarding tooth restoration integrity at 6-months vs. 12-months. However, a statistically significant difference in tooth restoration integrity between the 4 subgroups at 12-months (*P* = .049). Also, the hypersensitivity score, there was a statistically significant difference between the 4-time intervals (*P* < .001) and a statistically significant difference in surface area changes between the 4 subgroups.

**Conclusions:**

Selective removal of carious tissue and SMART restoration, combined with dental home and professional preventive measures every 3 months maintained the integrity of restorations in severely and moderately affected permanent molars up to 1 year.

**Trial registration:**

The study protocol was retrospectively registered on Clinical Trials (NCT05931822–05/ 07/2023).

**Supplementary Information:**

The online version contains supplementary material available at 10.1186/s12903-024-04860-z.

## Background

Molar incisor hypomineralization (MIH) is a challenging worldwide clinical condition. MIH is defined as a qualitative developmental defect of a systemic origin in the enamel of one or all first permanent molars with or without incisors involvement. The term “MIH” was proposed initially by the European Academy of Pediatric Dentistry (EAPD) [1, 2].

Nowadays, MIH is considered to be a very common dental defect spreading among children. The worldwide prevalence of MIH is estimated to be 13.5% globally, with 36.6% of the cases have affected incisors [3], and around 27.4% require clinical interventions [4]. Consequently, hypomineralized enamel suffers from post-eruptive breakdown and hypersensitivity making these teeth more liable to caries development and sensitivity [2, 5–8].

Minimally invasive dentistry (MID) especially alternative restorative technique (ART) depends on four important concepts which are; early diagnosis, caries risk evaluation of the oral and extraoral factors as the socioeconomic condition of the family, the idea of a dental home and diet, minimal cavity preparation and using biologically active materials and recent bonding systems [9]. The latest addition to the caries prevention tools is silver diamine fluoride (SDF) [10–12]. SDF could be the thread of hope for such cases, as it can remarkably reduce the hypersensitivity related to MIH and may increase the caries resistance in addition to following the key points of minimal invasive dentistry [13].

The null hypothesis of this study states that the treatment of hypomineralized first permanent molars with the SMART technique (SDF and high viscosity glass ionomer restorations (HVGI)) would significantly enhance their physical and biological properties in terms of tooth structure integrity, restoration survival and sensitivity. The aim of this study was to compare the restoration integrity, hypersensitivity and digital surface area changes after one year follow up in SMART versus HVGIR with routine fluoride application in treating different degrees of severities of hypomineralized first permanent molars in children.

## Methods

This study was a random controlled clinical trial, with a split-mouth design, performed in the pediatric dentistry department, Mansoura University, Egypt. It was conducted at the period from Sep 2020 to Dec 2022.

This study was ethically approved by the Faculty of Dentistry, Mansoura University (A05031219). The study was simply explained to the parent and the benefit for the children was pointed out and parent /child consent and assent were obtained before starting the treatment. The study protocol was registered on Clinical Trials (NCT05931822–3rd of July 2023).

Sample size was calculated by using Power Analysis and Sample Size (PASS) Software (2017). NCSS, LLC. Kaysville, Utah, USA. A factorial design with two factors (factor A is moderate and severe grades, and factor B is the SDF vs. Varnish) at 2 and 2 levels, respectively, has 4 cells to test their effects on restoration integrity. A total of 56 teeth are required to provide 14 teeth per cell. This design achieves 84% power when an F test is used to test factor A at a 5% significance level and the effect size is 0.400, achieves 84% power when an F test is used to test factor B at a 5% significance level and the effect size is 0.400, and achieves 84% power when an F test is used to test the A*B interaction at a 5% significance level and the effect size is 0.400 [14, 15].

### Diagnosing, training and calibration

Before starting the study, the examiner was thoroughly calibrated by using the EAPD scale under an expert supervision. Thirty pictures were used with different severities until a suitable intra-examiner reliability was attained through discussions and practical exercises. Molars which were presented with opacities and post eruptive breakdown restricted to the enamel, were judged to be moderate MIH. While, teeth erupted with breakdown involving dentin, were judged to be severe MIH [16].

### Study design

**Fig. 1:**
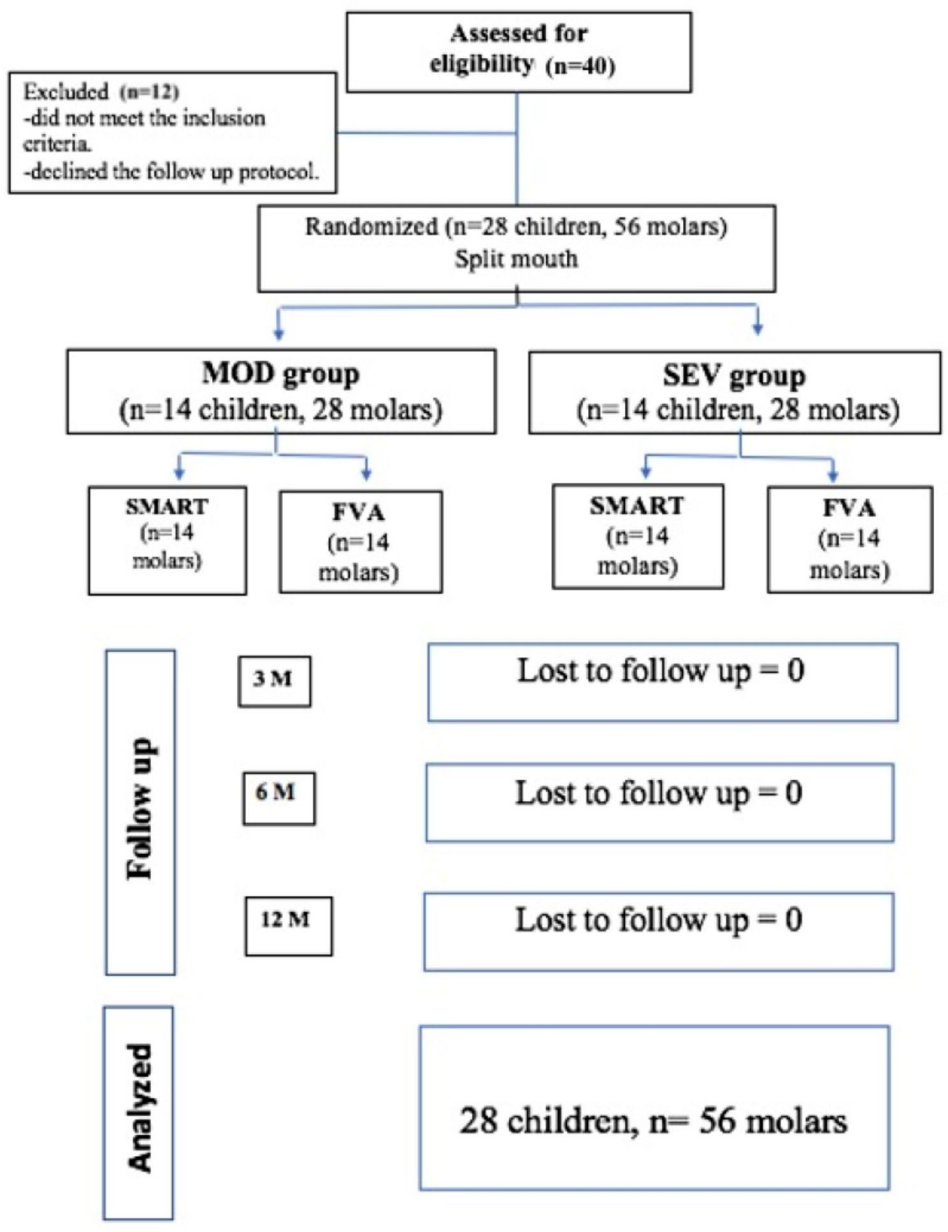
Flow diagram of participants up to 1 year

The study was conducted on 56 molars for 28 children (20 girls and 8 boys) with at least 2 hypomineralized molars with the same defect severity. The study comprised 2 groups; MOD group (moderate hypomineralized molars) and SEV group (severe hypomineralized molars) (*n* = 28 each). Each group was further subdivided into 2 subgroups; SMART subgroup 1 and conventional-HVGIR (CONV) subgroup 2 (*n* = 14 each) according to the technique of restoration. All children were successfully followed for 12 months with no complications as shown in Fig. 1.

### Setting and participants

After examining 80 children diagnosed as having hypo mineralized first permanent molars, 28 children met the following inclusion criteria:

- Children aged 6–10 years with moderate or severe hypomineralized first permanent molars.
- Presence of bilateral first permanent molars with the same degree of hypomineralization severity and ICDAS-II score (international caries detection and assessment system) [17] ranging from 3 to 5 for the MOD and SEV groups.
- Molars should be free of any symptoms and signs of irreversible pulpitis or pulp necrosis.
- Children should be free from any systemic diseases.

The rest of the children were excluded from the study due to having pulpal symptoms or lacking cooperation but were treated as indicated.

Children were examined clinically in a dental chair under the light source of the dental unit, using a dental oral mirror (No. 4). Teeth were cleaned before examination under wet conditions to better visualization. the modified European Academy of Pediatric Dentistry criterion was followed for defining MIH [18, 19].

### Randomization and blinding methods

Simple randomization was carried out through two principals: the restorative technique (SMART/CONV) and the site of the molar (left/right). To select the restoration and the tooth to be treated, each qualified child drew twice from four opaque envelopes. The first draw decided the restoration to be applied, while the second determined the site [20].

### Materials and clinical procedures

A split mouth design was performed for all groups, and each molar on either side was restored in a separate visit and local anesthesia was administered as needed. After that, hypomineralized enamel at the margins of the cavity was selectively removed using a high-speed, water- cooled diamond fissure burs. While at the enamel-dentin junction, a sharp sterile spoon double-ended excavator was used until hard and dry dentin remained with clean enamel margins. low-speed smart burs (SAMARTBURS II, RA-8) were used to remove the remaining caries in the depth of the cavity (pulpoaxial walls). Finally, dentine hardness was checked using a dental probe.

In subgroup 1, after caries removal, SDF (FAgamin 38%; Tedquim, Argentina) was applied in the cavity for 1 min then blot dried and the cavity was sealed with temporary filling. In the next visit after one-week, the temporary filling was removed and acidic conditioner was applied in the cavity for 10 s, washed with air water spray then dried with cotton pellets. Afterwards, the tooth was restored with HVGIR (Equia Forte HT; GC, Tokyo, Japan) following the instructions manual then covered by Vaseline. After setting time (2.5 min), the occlusion was checked with an articulating paper then adjusted.

In subgroup 2, caries was removed with the same technique, then the cavity was conditioned for 10 s then washed and dried with cotton pellets and restored by HVGIR. Finally, fluoride varnish (MI varnish; GC, Tokyo, Japan) was applied to all teeth of the child. Instructions were given to the children not to eat or drink for at least 1 h.

MI paste plus (MI paste plus; GC, Tokyo, Japan) was given to the children to use daily at night as tooth paste before going to sleep without rinsing. Oral hygiene measures instructions were illustrated to the parents/caretakers and children, with the use of auxiliary aids. Also, the brushing technique was demonstrated using toy models. Children were recalled every three months for fluoride varnish application on all teeth for one year.

### Evaluation

All children were evaluated for restoration and tooth integrity, hypersensitivity and occlusal surface area change.

**Fig. 2:**
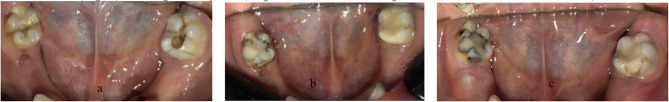
Severely affected first permanent molars treated with SMART (left) and conventional restoration (right) through one year (**a**: preintervention, **b**: postintervention, **c**: 12 months)

**Fig. 3:**
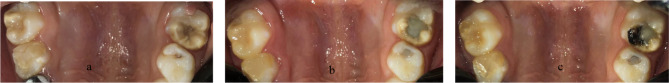
Moderately affected first permanent molars treated with SDF (left) and conventional restoration (right) through one year (**a**: preintervention, **b**: postintervention, **c**: 12 months)

The examiner did the evaluation after high reliable scores for restoration and tooth integrity. The standard ART criterion [21] was used for the evaluation, with codes from 0 to 9, in which 0, 1 and 7 are considered successful and rated “survived” while other scores rated as “failed to survive”. Photographs were taken postoperatively, at 6 months (T1) and at 12 months (T2) as shown in Figs. 2 and 3.

The Schiff Cold Air Sensitivity Scale (SCASS) was used to assess the molars hypersensitivity to air (0 = no response to the air; 1 = no response to the air, patient felt pain; 2 = response to air, patient moves away from air stimulus; 3 = response to the air, patient moves away from air stimulus and requests immediate discontinuation of the stimulus) [22].

Air was blown in a perpendicular position on the occlusal surface of the tooth using air syringe at a distance of 1 cm and if neighboring teeth were found, cotton rolls were used to cover them. Hypersensitive molars had a positive response to air stimulus when applied for 1 s, with a score of 2 or 3 [22].

For occlusal surface area change, two impressions were taken for the two groups and scanned at baseline and after 12 months. One step technique Impressions were taken for all groups twice, using additional silicone rubber base (Elite HD + putty soft and light, Zhermack, Italy), once immediately after restoring both molars then one-year after. Impressions were poured and casts were obtained for scanning using intraoral scanner (Medit i700) and analyzing the scans using Medit link 3.1.1 software program.

A 3-dimensional (3D) comparison was achieved by aligning the two scans for each impression by best fit alignment. A 3D comparison provides a heatmap with a spectrum of colors to show the differences between both scans, where the postoperative scan was selected as reference and the 12 months scan as test. The blue colour indicated the decrease in the vertical dimension and the red colour indicated an increase in the vertical dimension. A standard “best-fit alignment” uses an iterative closest point algorithm to align both scans that do not involve operator-based decisions [23, 24].

**Fig. 4:**
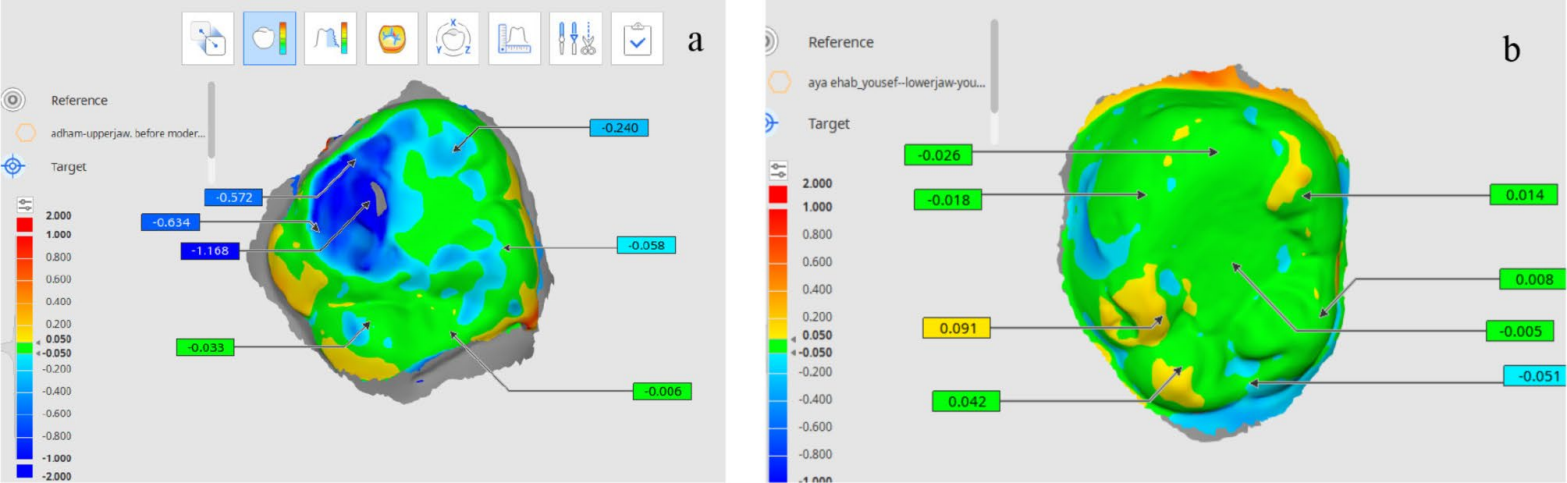
Heat map for different severities of MIH molars after matching both scans (postintervention and after one year) **a**: MOD/ SMART, **b**: SEV/CONV

The best fit alignment was done by creating a new modified reference area and the quality of the best fit was double checked using a 3D comparison. Quantitative measurements were obtained using the reference points for each first molar; mesiobuccal and distobuccal cusp tip, distal cusp tip, mesiolingual and distolingual cusp tip, central pit, mesial and distal marginal ridge as in Fig. 4.

### Statistical analysis

IBM/SPSS software (IBM Corp. Released 2020. IBM SPSS Statistics for Windows, Version 27.0. Armonk, NY: IBM Corp) was used to evaluate the data. Qualitative data were expressed as N (%) and Quantitative data were initially tested for normality using Shapiro-Wilk’s test with distributing the data normally if *p* > .050. Significant extreme values were tested by reviewing boxplots. Quantitative data were expressed as median and range (minimum – maximum).

Comparing the pre-post data was done using the Wilcoxon signed rank test as the difference was not normally distributed with ± significant outliers. While the non-normally distributed repeatedly measured quantitative data were compared using Friedman’s test. As for statistically significant difference, pairwise comparisons with Bonferroni correction for multiple tests were performed. The Kruskal-Wallis H-test was used to compare non-normally distributed quantitative data between groups. For statistically significant difference, pairwise comparisons with Bonferroni correction for multiple tests were performed. Results were considered as statistically significant if *P* ≤ .050 for all of the used tests. The results were graphically presented using proper charts whenever needed.

## Results

Twenty-eight children (20 girls and 8 boys) aged 6–9 participated in this study. A total of 56 molars were evaluated clinically for restoration integrity, hypersensitivity and surface changes. The recruitment of children along with the flow diagram are presented in Fig. 4. Molars distribution involved in the study is summarized in Table 1.

**Table 1:** Molars distribution

	Boys	Girls	Total
Maxillary molars	8 (14.3%)	16 (28.6%)	24 (42.9%)
Mandibular molars	13 (23.8%)	19 (33.3%)	32 (57.1%)
Total	21 (38.1%)	35 (61.9%)	56 (100%)

Regarding tooth restoration integrity, there was no statistically significant difference at 6-months vs. 12-months in each of the 4 subgroups. However, there was a statistically significant difference between the 4 subgroups at 12-months but not at 6-months. Pairwise comparisons with Bonferroni correction of significance values for multiple tests at 12-months revealed no statistically significant difference for any pair (p-value = 0.285 for MOD/SMART vs. SEV/ SMART, MOD/SMART vs. SEV/CONV, MOD/CONV vs. SEV/CONV, and MOD/ CONV vs. severe SMART (p-value = 1.000) and for MOD /SMART vs. MOD/CONV and SEV/ SMART vs. SEV /CONV) (Table 2).

**Table 2:** Tooth and restoration integrity in each subgroup over 1 year

Subgroup	Postintervention 6-months	Postintervention 12-months	Z	p-value
MOD/SMART	1 (1–1)	1 (1–2)	-1.414	0.157
MOD/CONV	1 (1–1)	1 (1–2)	-1.414	0.157
SEV/SMART	1 (1–1)	1 (0–1)	-1.414	0.157
SEV /CONV	1 (1–1)	1 (0–1)	-1.414	0.157
H [3]	0.000	7.857		
P-value	1.000	0.049		

*Notes* Data is median (minimum – maximum). The test of significance is Wilcoxon’s signed rank test which compares readings at 6- and 12-months in each group. The test of significance is Kruskal-Wallis H-test which compares readings between the 4-groups at both 6- and 12-months

**Fig. 5:**
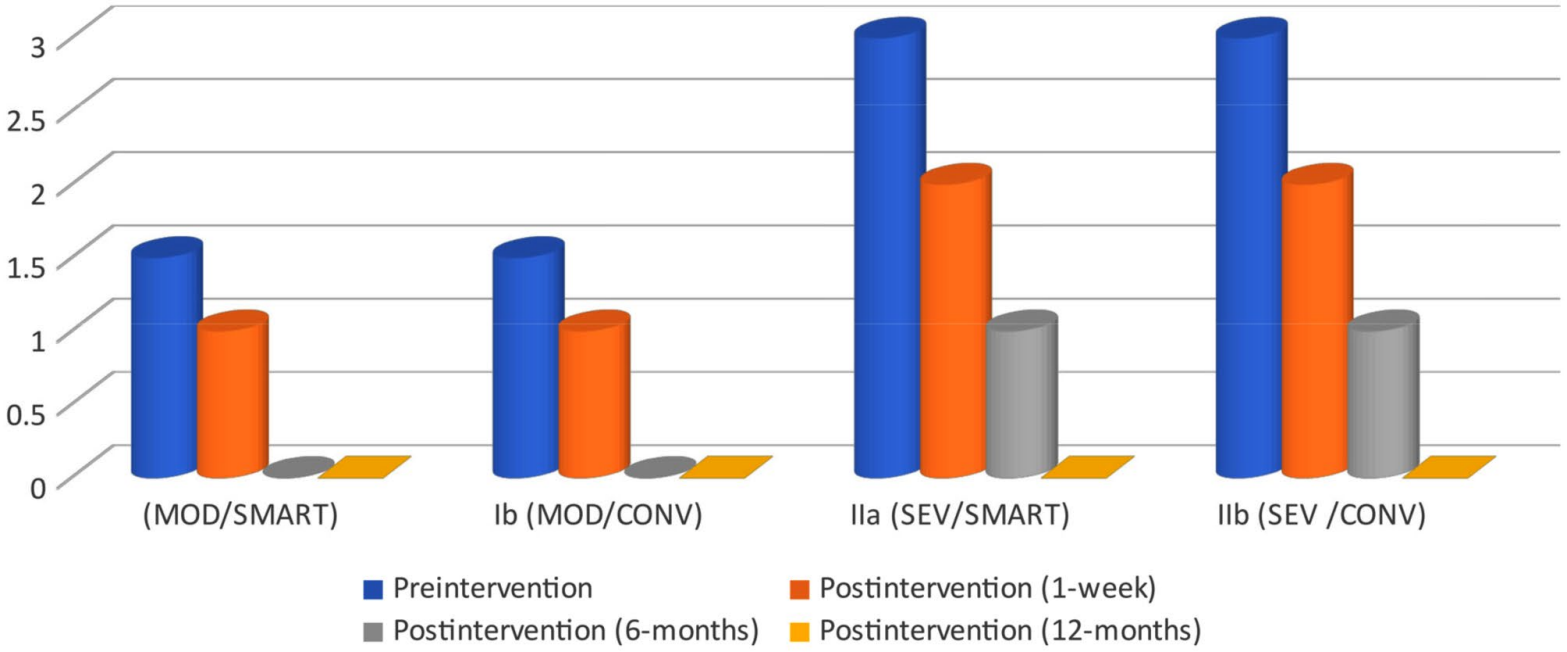
Hypersensitivity score (0–3) in each of the 4 groups over time

Table 3 shows a statistically significant difference in hypersensitivity score between the 4 time points in the moderate and severe subgroups as shown in Fig. 5.

**Table 3:** Hypersensitivity score (0–3) in each of the 4 groups over time

Group	Time				Test of significance	
	Preintervention	Postintervention (1-week)	Postintervention (6-months)	Postintervention (12-months)	χ^2^ [2]	p-value
MOD/SMART	1.5 (1–3)	1 (1–1)	0 (0–0)	0 (0–0)	40.765	< 0.001
MOD/CONV	1.5 (1–3)	1 (1–1)	0 (0–0)	0 (0–0)	40.765	< 0.001
SEV/SMART	3 (3–3)	2 (2–2)	1 (1–1)	0 (0–0)	42.000	< 0.001
SEV /CONV	3 (3–3)	2 (2–2)	1 (1–1)	0 (0–0)	42.000	< 0.001

*Notes* Data is median (minimum – maximum). The test of significance is Friedman’s test. Significance values have been adjusted by the Bonferroni correction for multiple tests

Accordingly, pairwise comparisons are presented in Table 4. This table shows no statistically significant difference in hypersensitivity score between preintervention vs. one-week and between 6-months and 12-months intervention in each of the 4 moderate and severe subgroups, and between one-week vs. 6-months in the severe subgroups. Hypersensitivity scores showed statistically significantly difference between preintervention vs. 6-months, and 12-months and between 1-week vs. 12-months in each of the 4 moderate and severe subgroups, and between 1-week vs. 6-months in the two moderate subgroups (Table 5).

**Table 4:** Pairwise comparisons for hypersensitivity score

Group	Preintervention vs. 1-week	Preintervention vs. 6-months	Preintervention vs. 12-months	1-week vs. 6-months	1-week vs. 12-months	6-months vs. 12-months
MOD/SMART	1.000	< 0.001	< 0.001	0.002	0.002	1.000
MOD/CONV	1.000	< 0.001	< 0.001	0.002	0.002	1.000
SEV/SMART	0.243	< 0.001	< 0.001	0.243	< 0.001	0.243
SEV /CONV	0.243	< 0.001	< 0.001	0.243	< 0.001	0.243

*Notes* Significance values have been adjusted by the Bonferroni correction for multiple tests

**Table 5:** Comparisons of surface area changes

**Subgroup**	Surface area change (mm^2^)			H [3]	p-value
	Median	Minimum	Maximum		
MOD/SMART	39.3	36.1	41.4	34.924	< .001
MOD/CONV	42.4	40.1	50.7		
SEV/SMART	40.7	37.2	43.2		
SEV/CONV	46.0	42.2	56.4		

*Notes* The test of significance is Kruskal-Wallis H-test

This table shows a statistically significant difference in surface area changes between the 4 subgroups.

### Pairwise comparisons

Statistically significant difference was found using Kruskal-Wallis H-test, so pairwise comparisons with Bonferroni correction for multiple tests were run and showed that there was a statistically significant difference between MOD/SMART vs. MOD/CONV group (p-value = 0.008), between MOD/SMART vs. SEV/CONV (*p* < .001), and SEV/SMART vs. SEV/CONV (*p* < .001), but not between MOD /SMART vs. SEV/SMART (p-value 1.00), SEV/SMART vs. MOD/CONV (*p* = .244), or between MOD vs. SEV/CONV (*p* = .138).

## Discussion

The success of the restorative treatment mostly depends on the lesion severity, as well as the child’s cooperation, age and hygiene habits [25]. Also, the material and the restorative technique used plays an important role.

One of the goals of the study was to evaluate the success rate of resin modified glass ionomer restorations using a SMART restorative technique in hypomineralized first permanent molars. Also, to assess the effect of SDF on the hypersensitivity accompanying this type of defect. High success rates were displayed in the results after 12 months, proving that such technique is a practical option for managing hypomineralized first permanent molars in children.

The age in this study was chosen from 6 to 9, in order to follow the first permanent molar from the beginning of its eruption. Sometimes in MIH cases, it even erupts earlier than 6 years of age. Moreover, at this age most children would have had all four first permanent molars. At an older age, there would be a higher risk of PEB, caries initiation and later pulp involvement [26]. A split mouth design was performed for standardization and subjecting both molars to the same oral hygiene measures and environment.

It was perceived in the study that mandibular molars were more affected than maxillary molars, which might be due to gravity as food stagnates normally more on lower teeth. Also, girls were more affected than boys.

Minimally invasive technique was adopted in this study to avoid further destruction to already weakened molars and to help children perform proper oral hygiene to enhance their quality of life [27, 28].

Thermal and mechanical stimuli cause sensitivity to hypomineralized teeth, which may cause child distress while performing the simple form of oral hygiene, for this specific reason, SDF was used in order to decrease the hypersensitivity of such teeth. Additionally, in severe types of MIH, molars are more liable to restoration failure with recurrent interventions [29, 30].

From the merits of SMART restorations their simplicity, lesser operational time, decreased sensitivity and less behavioral dilemmas in MIH compared to conventional caries removal [31, 32]. Crystal YO et al. stated in their study that the use of 38% SDF can improve the management of dental caries programs between groups [33].

Grossi et al. [9] reported a very high rate of survival (98%) of MIH first permanent molars restored with HVGI restorations via atraumatic restorative technique, which was comparable to the current study results.

The reason behind SMART subgroups being stronger with less surface deformities, is probably attained to its ability in arresting caries, due to the action of silver, which has a bactericidal action against cariogenic bacteria, along with the high fluoride concentration which is retained in enamel and dentine 2–3 times more than topically applied 5% fluoride varnish, which was in accordance with Burgess et al. [34].

In moderate group, it was noticed that, the tooth integrity after restoration to be less than that of severe group but it was clinically nonsignificant, which is hard to comprehend, but this can be explained by the fact that in severe cases most of the tooth structure is replaced by restoration, which in this case is harder than the actual tooth structure in hypomineralized molars. In addition, in moderate group, part of the tooth was restored, and most of the deteriorations seen were not in the restoration, but in the actual surrounding tooth structure.

Comparable results were concluded by Hernandez et al. [35]. They performed similar procedures on hypomineralized molars and failures appeared due to enamel fracture at the margins of the restorations after 2 years of follow up.

Restoration of such molars have a great effect on decreasing the hypersensitivity regardless of the treatment used, whether it is SDF with HVGI or even HVGI alone.

The current study results of hypersensitivity were comparable to the results obtained by Ballikaya et al. [14] Both studies noticed a decrease in the hypersensitivity after the first intervention with SDF. The present study revealed that, the annual application of SDF and the regular fluoride varnish application had a very comparable similarity in results. This may be due to the change of the biofilm through the whole oral cavity, owing to SDF application to one molar, as in some cases during treatment some un treated cavities turned black even we did not apply SDF to them [14].

One of the prominent results is that there was a significant difference between SMART and conventional in the severe group regarding the surface area change, as the change in SMART subgroup was less than conventional. This may be due to the effect of SDF increasing the strength of tooth structure making it harder. In a study by Dos Santos on primary molars comparing between SDF and glass ionomer restorations, molars treated with SDF had 85% of their caries arrested and their dentine was hard, where those in the Interim restorative treatment group had lost more than 40% of their Glass ionomer, which was comparable to the current study [36].

Chu et al. in their study had comparable results after using MI paste plus, as it contains CCP-ACP (Casein phosphopeptide–amorphous calcium phosphate), which was found to enhance the physical strength of the MIH-affected enamel, which was likely due to an increase of the mineral content [37]. Cardoso et al. concluded in their study, that the mineral density was improved and even the arrangement of the enamel rods was enhanced after treatment with CPP-ACP tooth mousse [38]. These results augment the findings in the current study.

The cause behind the comparable results between SMART and conventional treatment in the moderate group may be regarded to the fact that in moderate group, the occlusal surface consists of restoration material and hypomineralized tooth structure unlike the severe group, where most of the occlusal surface is restored.

Moreover, it was noticed clinically in the moderate conventional subgroup that the surrounding tooth structure to the restoration was less integrate than the restoration itself, unlike the SMART which encountered less tooth structure disintegration, again this can be correlated to the fact of SDF increasing the mineral density of dentine and enamel.

This study showed some limitations; firstly, is the patterns of the cavities in hypomineralized molars, which should be given thought to, especially how much affected enamel should be removed. Second, is the scanning of the casts, it would have been better to scan the molars directly with an intraoral scanner for more accurate results. Unfortunately, it was not available to get the scanner where the study was performed. Finally, superimposing 3D scans can be prone to errors which needs some technique modification.

This study seems to be one of a few clinical trials evaluating the longevity of silver modified HVGI restorations in severely and moderately affected hypomineralized permanent molars. The treatment revealed acceptable survival rates at the 12-months follow-up, proposing the effectiveness of this minimally invasive technique in hypomineralized permanent molars. These results conclude mainly that the management of hypomineralized permanent molars in children should follow a more conservative approach [39]. Nevertheless, the long-term effects of treatment should be evaluated in forthcoming trials along with evaluating the oral flora before and after SDF application.

## Conclusions

Within the limitations of this study, it can be concluded that:

- Selective caries removal and clean enamel margins restored with SMART restorations, combined with professional preventive measures every 3 months maintained the integrity of restorations in permanent molars with severe and moderate MIH for up to 1 year.
- In severe MIH, SMART restorations can be an alternative choice till the complete eruption of first permanent molar.
- SDF and fluoride varnish have a significant and comparable impact on the decrease of hypersensitivity in MIH molars.
- Surface area changes in SMART subgroups were less than conventional subgroups.

## Electronic supplementary material

Below is the link to the electronic supplementary material.

Supplementary Material 1

## Data Availability

The datasets generated and/or analyzed during the current study are not publicly available [as it is not published yet], but are available from the corresponding author on reasonable request.

## Abbreviations

SMART: Silver diamine fluoride modified atraumatic restorative treatment
MIH: Molar incisor hypomineralization
EAPD: European Academy of Pediatric Dentistry
MID: Minimally invasive dentistry
ART: Alternative restorative technique
SDF: Silver diamine fluoride
HVGIR: High viscosity glass ionomer restorations
PASS: Power Analysis and Sample Size
ICDAS-II: International Caries Detection and assessment system
SCASS: Schiff Cold Air Sensitivity Scale
CCP-ACP: Casein phosphopeptide–amorphous calcium phosphate
